# Causes and trends of adult mortality in southern Ethiopia: an eight-year follow up database study

**DOI:** 10.1186/s12879-023-07988-5

**Published:** 2023-01-18

**Authors:** Gebrekiros Gebremichael Meles, Gistane Ayele, Befikadu Tariku Gutema, Mekides Kondale, Zerihun Zerdo, Behailu Merdekios, Tsegaye Tsalla, Mesfin Kote, Alazar Baharu, Alemayehu Bekele, Feleke Gebremeskel, Mulugeta Shegaze, Teklemariam Gultie

**Affiliations:** 1 School of Public Health, College of Medicine and Health Sciences, Arba Minch University, Arba Minch, Ethiopia; 2 Department of Medical Laboratory Sciences, College of Medicine and Health Sciences, Arba Minch University, Arba Minch, Ethiopia; 3 Department of Computer Sciences, Arba Minch University, Arba Minch, Ethiopia; 4 Ethiopian Public Health Association, Addis Ababa, Ethiopia; 5 Department of Midwifery, College of Medicine and Health Sciences, Arba Minch University, Arba Minch, Ethiopia; 6 School of Public Health, College of Health Sciences, Mekelle University, Mekelle, Ethiopia; 7 School of Public Health, Cheeloo College of Medicine, ShandongUniversity, Jinan, Shandong 250012, China

**Keywords:** Adult mortality, Causes of adult death, Arba Minch HDSS

## Abstract

**Background:**

Broad and specific causes of adult mortalities are often neglected indicators of wellbeing in low-income countries like Ethiopia due to lack of strong vital statistics. Thus, this database study aimed to assess the causes of adult mortality using demographic surveillance data.

**Methods:**

An 8-year (12 September 2009–11 September 2017) surveillance data from the Arba Minch Health and Demographic Surveillance Site was used for this study. Verbal autopsy methods and ICD codes were used to identify the causes of the adult deaths. The collected data were entered to the database by data clerks. We used Microsoft Excel and STATA version 16 software for data cleaning and analysis. Chi-squared test was used to see the significances of the trend analyses.

**Result:**

From the 943 adult deaths from 2009 to 2017 in the Health and Demographic Surveillance Site in southern Ethiopia, more than half of them were females. The specific leading cause of death in the adults were tuberculosis (16.8%), malaria (9.7%), and intestinal infectious diseases (9.6%).

Communicable diseases (49.2%, 95% C.I 45.7, 52.7) accounted for about half of the deaths followed by non-communicable diseases (35%, 95% C.I 31.7, 38.4) where both categories showed an increasing trend.

**Conclusion:**

Although pieces of evidences are showing the shift from communicable diseases to non-communicable diseases as the major causes of adult death in developing countries, this study showed that communicable diseases are still the major causes of adult deaths. Efforts and emphasis should be given to control infectious diseases such as tuberculosis and malaria.

**Supplementary Information:**

The online version contains supplementary material available at 10.1186/s12879-023-07988-5.

## Background

Globally, approximately 55.4 million people died in 2019 [1]. The global share of the deaths due to Non-communicable diseases (NCDs) such as cardiovascular diseases, cancers, diabetes, and chronic lung diseases increased from almost 61% in 2000 to 73.6% in 2019 [2, 3] followed by communicable, maternal, neonatal, and nutritional conditions (18.4–20%) and injuries (8–9%) [2, 4, 5]. There is a decreasing rate for communicable diseases and injuries [5].

According to the World Health Organization (WHO) Africa region, the overall adult mortality of the region was 308 per 1000 population, and in Ethiopia, it was 218, 239, and 198 per 1000 population for both sexes, males and females respectively in 2015 [6]. This is the only WHO region where still the leading causes of death (52.9–56%) are the communicable, maternal, neonatal, and nutritional conditions killing 2.3 million people in 2016 [2, 4, 7].

Evidence from International Network for the Demographic Evaluation of Populations and Their Health (INDEPTH) Health and Demographic Surveillance System sites from Asia and Africa reported that a mortality rate of 10.9 per 1000 person-years whereby 35.6% of the deaths were due to Non-Communicable Diseases [8]. A triple burden of infectious diseases, chronic diseases, and external injuries is still resulting in modest adult mortality level decline in most African countries except in north Africa in the last few decades [7].

A paradoxical kind of adult mortality trends is documented in findings from low- and middle-income countries. Decreasing adult mortality rates are evidenced in studies from South Korea, Bangladesh, Gambia, Ghana, and South Africa [9–13]. On the contrary, increasing mortality trends were documented elsewhere [14–16]. Despite the substantial improvements in adults’ survival in some countries in sub-Saharan Africa, still, this region has the heaviest burden of adult mortality worldwide [17]. In the northern part of Ethiopia, it is reported that the non-communicable diseases account for 36.4% of the total deaths followed by communicable disease (34.9%) [18].

So far, in sub-Saharan African countries including Ethiopia, more studies had focused on child and maternal mortalities, whereas a little emphasis is given to causes of adult mortalities [19]. The mortality estimation for this population is impeded by the lack of data and due to discrepant estimates [7, 20]. Estimating the causes of adult mortality is more difficult in countries without strong vital statistics [21, 22]. This study aimed to identify the causes of adult mortality in the Arba Minch health and demographic surveillance system.

## Methods

### Study setting

The study used surveillance data conducted in Arba Minch Health and Demographic Surveillance Site (Arba Minch HDSS) which was established in 2009 in collaboration with Arba Minch University. Nine rural and one urban kebeles (administrative units below districts) were intentionally selected as catchment area Based on climatic zone two of the kebeles are highlands, four lowlands and the rest three midlands. The main objective of Arba Minch HDSS is to collect longitudinal data on birth, death, and migration in the selected kebeles of Arba Minch Zuria district [23].

The Arba Minch HDSS site follows-up every individual within a defined catchments area two times a year with house-to-house visits. During the visits, if deaths happened, they register and collect information about the cause of death by using the standard WHO verbal autopsy questionnaires [24]. The design of the surveillance is population-based longitudinal follow up, and this study used the data from September 2009 to September 2017 to identify the causes of adult mortality in the surveillance site.

### Data collection procedures

The Arba Minch Health and Demographic surveillance system use a verbal autopsy technique to identify the cause of deaths. Verbal autopsy is a technique used to determine the cause of death by asking caregivers, friends, or family members about signs and symptoms exhibited by the deceased in the period before death. This is done using a standardized questionnaire that collects details on signs, symptoms, complaints, and any medical history or events [24].

The occurrence of death in the demographic surveillance area was notified by the local village-based data collectors and guides. The causes of death were ascertained based on an interview with next of kin or other caregivers using a standardized questionnaire that draws information on signs, symptoms, medical history, and circumstances preceding death after 45 days mourning period. On the agreed day, the VA interviewer arrived at the residence of the deceased to interview with the person who was responsible for caring for the deceased. In the case of the absence of an appropriate interviewee, up to three attempts were made to conduct an interview.

VA data collectors would make sure that every section of the form would be accurately completed before the form submitted to field supervisors for scrutiny of the quality of the collected data. The completed VA questionnaires were given to two blinded physicians and reviewed independently. When disagreements in diagnosis arose, a third physician was assigned to review the case. The final diagnosis was assigned based on the agreement between the third physician and any of the two physicians. The case was considered ‘undetermined’ if all three physicians assigned a different diagnosis. Physicians label the death as ‘unspecified causes of death (VA-99)’ when it was difficult to classify based on the given information. Two physicians, trained in VA diagnosis and coding procedures assigned codes and titles for each cause of death as underlying, immediate, and contributing factors independently using the information in VA forms based on WHO International Classification of Diseases-10 and VA code system [25].

### Classification of causes of death

We have used the following classifications of causes of death for this study based on the international disease classification system [26, 27].

#### Communicable diseases (CDs)

All infectious and parasitic diseases including human immunodeficiency virus (HIV), tuberculosis, malaria, intestinal infection, infectious diseases of an unspecified cause, acute lower respiratory infections, meningitis, viral hepatitis, typhoid and paratyphoid fever, and rabies.

#### Non-communicable diseases (NCDs)

Diseases of the circulatory system, neoplasms, renal disorders, respiratory disorders, gastrointestinal disorders, mental, and nervous system disorders and nutritional and endocrine disorders.

#### External causes of death (ECs)

Accidental falls, accidental drowning and submersion, burn, intentional self-harm, and others that are not related to the above two categories.

#### Pregnancy, childbirth and puerperium

All deaths related to pregnancy, childbirth, and postpartum such as maternal deaths associated with abortion, childbirth-related hemorrhage.

### Data analysis procedures

The data were entered into an excel database system by the data clerks of the Arba Minch HDSS. Data cleaning and analysis was done using STATA 16 software and Microsoft Excel. Description of the adult deaths was made by various sociodemographic characteristics such as sex, residence, age category, marital status, occupation, educational status, and place of death.

Both the specific and broad causes of death among the adults aged 15 years and above were identified according to the verbal autopsy diagnoses. We excluded deaths with discordant verbal autopsy diagnoses, unspecified cause of death, and deaths without verbal autopsy diagnosis from the denominators in the calculation of the proportions of each cause of deaths. We have also used a chi-squared test to compare some specific causes of death among different sociodemographic characteristics, and to test the trends in major causes of death.

The analyzed data is from September 2009 to September 2017. But for the purpose of analysis and comparison, we categorized the death years into eight equal categories (i.e. 1 year) as follow;

- Year 1: 12 September 2009 to 11 September 2010.
- Year 2: 12 September 2010 to 11 September 2011.
- Year 3: 12 September 2011 to 11 September 2012.
- Year 4: 12 September 2012 to 11 September 2013.
- Year 5: 12 September 2013 to 11 September 2014.
- Year 6: 12 September 2014 to 11 September 2015.
- Year 7: 12 September 2015 to 11 September 2016.
- Year 8: 12 September 2016 to 11 September 2017.

## Results

### Socio-demographic characteristics

A total of 943 adult deaths were recorded in the Arba Minch-HDSS in the eight surveillance periods (from September 2009 to September 2017). Accordingly, 54% of them were females, and about 62% were married. The majority of the deceased persons were from rural residence (88.85%), aged between 55 and 74 years (32.8%), unable to read and write (96.78%) by educational status, and farmers (45%) by occupation. The median ± interquartile range age of the deceased was 54 ± 38. Regarding the place of death, 736 (78%) individuals died at home (Table 1).

**Table 1:** Sociodemographic characteristics of the deceased from 2009 to 2017, Arba Minch HDSS

Characteristics	Frequency (n = 943)	Percent
Sex		
Male	509	46
Female	434	54
Age group		
15–24	123	13
25–34	130	13.8
35–44	106	11.26
45–54	120	12.74
55–64	151	16
65–74	158	16.8
75–84	94	10
85+	60	6.4
Age (median ± IQR)	54 ± 38	
Residence (n = 942)		
Rural	837	88.85
Urban	105	11.15
Marital status		
Married	588	62.35
Single	139	14.74
Widowed	193	20.47
Other	23	2.44
Educational status		
Unable to read or write	658	96.78
Primary education	220	23.33
Secondary education and above	65	6.89
Occupation		
Housewife	236	25
Farmer	425	45
Daily laborer	73	7.74
No work	81	8.6
Other*	128	13.57
Place of death		
Home	736	78
Health institutions	143	15.2
Other	64	6.8

*Merchant, government employee, students

Almost half (49.6%) of the deaths occurred in lowland followed by highland (38.2%) and midland (12.2%) areas of the surveillance site according to climatic conditions.

### Causes of adult deaths

Among the total deaths, specific and broad causes of death were identified from the Verbal Autopsy (VA) for 924 cases. The final VA code was missing for 19 cases and discordant for 79 cases. Besides, the cause of death for 65 (8.8%) of the deaths was unspecified, based on the VA questionnaire (VA-99 code). A total of 75 VA codes of specific causes of deaths were recorded for the above deaths.

#### Broad causes

The causes of deaths were categorized into communicable diseases (all infectious and parasitic diseases), non-communicable diseases (diseases of the circulatory system, nervous system, nutritional diseases, renal disorders, respiratory disorders, and gastrointestinal disorders), pregnancy, child, birth, and postpartum related causes, and external causes of death. Accordingly, about half of the deaths were attributed due to communicable diseases (49.2%, 95% C.I 45.7, 52.7) followed by non-communicable diseases (35%, 95% C.I 31.7, 38.4), external causes of death (13.6%, 95% C.I 11.2, 16), and pregnancy, child, birth and postpartum related causes (2.2%, 95% C.I 1.2, 3.23).

**Fig. 1:**
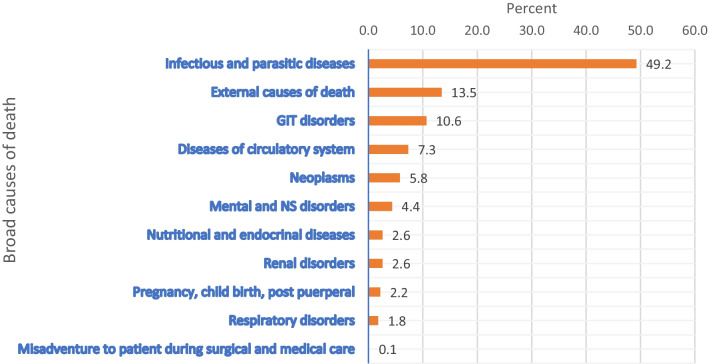
Broad causes of death from 2009 to 2017, Arba Minch HDSS

The leading broad causes of death in the surveillance site was infectious and parasitic diseases (49.2%, 95% C.I 45.7, 52.7) followed by external causes of death (13.5%, 95% C.I 11.1, 15.9). There was a single death assumed to be attributed due to misadventure to a patient during surgical and medical care (Fig. 1).

**Fig. 2:**
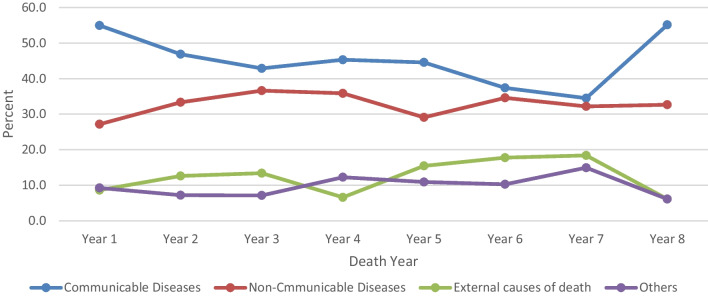
Trends of the broad causes of adult deaths from 2009 to 2017, Arba Minch HDSS

Although the total number of deaths from all causes shows a decreasing trend, the percent share of deaths in each surveillance year showed an increasing trend for communicable diseases compared to the other broad causes of adult deaths (Fig. 2).

**Fig. 3:**
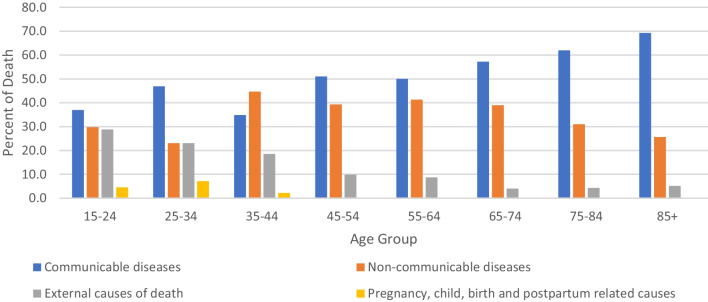
Broad causes of death by age category of the deceased from 2009 to 2017, Arba Minch HDSS

Furthermore, the distribution of the broad causes of death was classified by the age category of the deceased. In the majority of the age categories, the communicable diseases group accounts for the majority of the deaths followed by non-communicable diseases (Fig. 3).

**Fig. 4:**
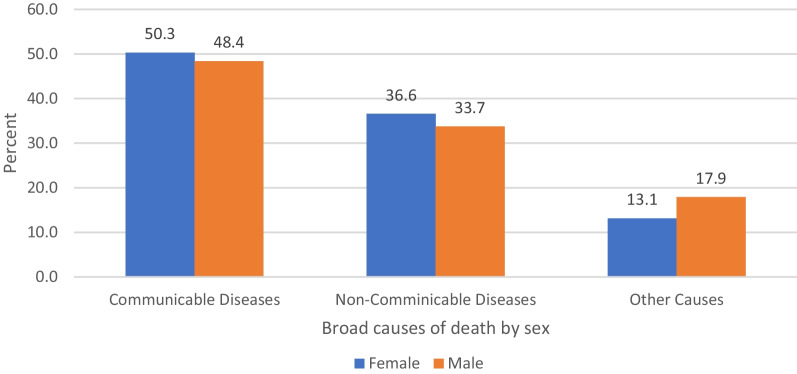
Broad causes of death by sex of the deceased from 2009 to 2017, Arba Minch HDSS (chi-squared test = 3.4, P-value = 0.2)

The distribution of the broad causes was done by the sex of the deceased. Accordingly, the communicable group of diseases tends to be the leading cause of death in both females and males. Actually, there was no statistically significant association in the distribution of causes of deaths among males and females (Fig. 4).

#### Specific causes of death

Among the specific causes of death in the study area, tuberculosis was the commonest (16.8%, 95% C.I 14.2, 19.4) single cause followed by malaria (9.7%, 95% C.I 7.6, 11.8), intestinal infectious diseases (9.6%, 95% C.I 7.5, 11.8), and chronic liver disease (5.6%, 95% C.I 4, 7.2). Among the commonest specific causes of adult deaths, diseases such as malaria, intestinal infectious deaths, chronic liver diseases, and intentional self-harm caused more deaths in males than females. On the contrary, congestive heart failure, cardiovascular diseases, and typhoid and paratyphoid caused more female deaths than males. A nearly similar distribution of deaths among males and females were observed from tuberculosis, HIV/AIDS, and gastric and duodenal ulcer (Table 2).

**Table 2:** The top twenty specific causes of death by sex from 2009 to 2017, Arba Minch HDSS

S.no.	Disease	Frequency (n = 780)		Total (%)
		Male (%)	Female (%)	
1	Tuberculosis	64 (48.9)	67 (51.1)	131 (16.8)
2	Malaria	47 (61.8)	29 (38.2)	76 (9.7)
3	Intestinal infectious diseases*	44 (58.7)	31 (41.3)	75 (9.6)
4	Chronic liver diseases	35 (79.5)	9 (20.5)	44 (5.6)
5	HIV/AIDS	20 (48.8)	21 (51.2)	41 (5.3)
6	Acute lower respiratory infections**	11 (45.8)	13 (54.2)	24 (3.1)
7	Intentional self-harm	18 (75)	6 (25)	24 (3.1)
8	Epilepsy	15 (71.4)	6 (28.6)	21 (2.7)
9	Accidental fall	14 (66.7)	7 (33.3)	21 (2.7)
10	Assault	14 (70)	6 (30)	20 (2.6)
11	Congestive heart failure	5 (27.8)	13 (72.2)	18 (2.3)
12	Gastric and duodenal ulcer	8 (50)	8 (50)	16 (2.1)
13	Severe malnutrition	10 (66.7)	5 (33.3)	15 (1.9)
14	Hypertensive diseases	9 (64.3)	5 (35.7)	14 (1.8)
15	Cardio vascular diseases	6 (42.9)	8 (57.1)	14 (1.8)
16	Renal failure	8 (57.1)	6 (42.9)	14 (1.8)
17	Other transport accident	8 (72.7)	3 (27.3)	11 (1.4)
18	Neoplasm of uncertain behavior, unspecified	5 (50)	5 (50)	10 (1.3)
19	Other diseases of the intestine	4 (40)	6 (60)	10 (1.3)
20	Typhoid and paratyphoid	3 (33.3)	6 (66.7)	9 (1.2)

*Including diarrheal diseases

**Including pneumonia and acute bronchitis

**Fig. 5:**
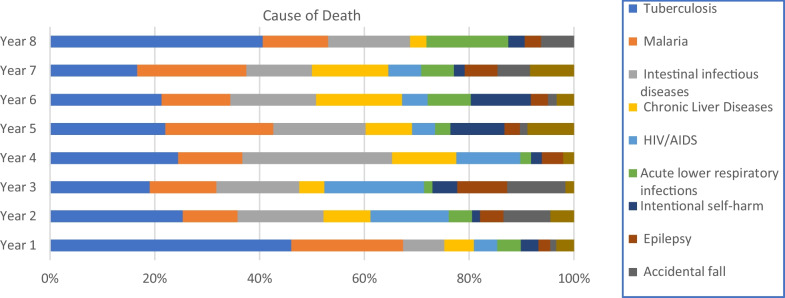
Trends of the ten tops specific causes of adult deaths from 2009 to 2017, Arba Minch HDSS

**Fig. 6:**
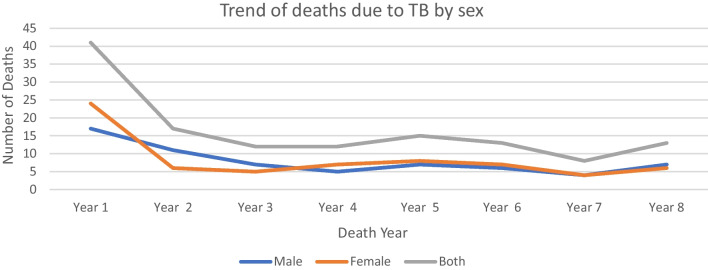
Trends of adult deaths due to tuberculosis by sex from 2009 to 2017, Arba Minch HDSS (*Both sexes: χ*^*2*^ *test for trend = 4.65, P-value = 0.03, Male: χ*^*2*^ *test for trend = 1.24, P-value = 0.27, Female: χ*^*2*^ *test for trend = 2.61, P-value = 0.11*)

Although the mortality trend from tuberculosis was declining from year to year, it showed a dramatic increase in 2017. On the other hand, the trend of adult mortality from malaria showed a steady decrement. Similarly, the trend of adult deaths due to tuberculosis was decreasing (χ^2^ test for trend = 4.65, P-value = 0.03). However, a nearly identical trend was observed for both males and females (Figs. 5, 6).

## Discussion

In this study, the major causes of adult mortality were identified. The trends of adult mortality in the demographic surveillance site were also examined. The majority of the deceased were females, from rural residence, aged between 55 and 74 years, unable to read and write, and farmers from 2009 to 2017 in Arba Minch Health and Demographic Surveillance Site. Almost similar proportions in the above socio-demographic characteristics excepting the age group were observed in studies conducted in Ethiopia and other African settings [18, 28, 29].

This study showed that the leading broad causes of death in the surveillance site were infectious and parasitic diseases (49.2%) followed by external causes of death (13.5%), gastrointestinal disorders (10.6%), disease of the circulatory system (7.3%), and neoplasms (5.8%). Similar findings were documented showing communicable diseases as the major causes of adult deaths in various African countries [10, 28, 30–33]. This finding is in contrary to a study done in the northern part of Ethiopia, where the non-communicable diseases were the major killers [34]. Such discrepancies may be due to differences in socioeconomic status, and lifestyles of the community which play an important role in the development of the NCDs.

Among the specific causes of death in the study area, tuberculosis was the commonest (16.8%) single cause followed by malaria (9.7%), intestinal infectious diseases (9.6%), and chronic liver disease (5.6%). In line with this finding, tuberculosis is the single common cause of death in different parts of Ethiopia and other sub-Saharan African countries [10, 18, 28, 30, 31]. This indicates that tuberculosis is still the single most cause of mortality of Ethiopian adults.

Although an increasing trend of tuberculosis incidence was documented elsewhere in Ethiopia [27, 35], the adult deaths due to tuberculosis are steadily decreasing in this study for both males and females. This may be due to a relatively better diagnosis and treatment of tuberculosis in health facilities currently in Ethiopia. Similarly, the trend of adult mortality from malaria showed a steady decrement in this study, although a declining bed net utilization among pregnant mothers is reported from similar area [36]. The declining trend of death from malaria, does not however, show a low malaria prevalence in the area, as it is still the second most cause of adult deaths.

### Limitations

One of the major limitations of this study is that we only described the causes of adult mortality failing to identify the possible risk factors. We also missed the verbal autopsy result for 19 cases. Another limitation is the validity of physician certified verbal autopsy to identify the causes of adult deaths as it may yield biased results. Not including the data after 2017in the analysis may be also taken as a limitation of this study. The pattern of the cause of death maybe skewed to communicable diseases as majority of the study participants are from rural.

## Conclusion

Tuberculosis is still the leading cause of adult mortality in the rural part of southern Ethiopia using VA. Although pieces of evidences are showing the shift from communicable diseases to non-communicable diseases as the major causes of adult death in developing countries including Ethiopia, this study showed that communicable diseases alone account for about half of the adult deaths.

## Supplementary Information

**Additional file 1.** Full peer review history.

## Data Availability

The data that support the findings of this study are available from the Arba Minch HDSS database, but restrictions apply to the availability of these data and so are not publicly available. Data are, however, available from the authors upon reasonable request and with permission of the HDSS coordinator.

## Abbreviations

AIDS: Acquired Immune-Deficiency Syndrome
CDC: Center for Diseases Control
EPHA: Ethiopian Public Health Association
HDSS: Arba Minch Health and Demographic Surveillance Site
NCD: Non-communicable diseases
TB: Tuberculosis
VA: Verbal autopsy
WHO: World Health Organization
