# Supplementary material for: Causes and trends of adult mortality in southern Ethiopia: an eight-year follow up database study
Source: BMC Infect Dis. 2023 Jan 18;23:29. doi: 10.1186/s12879-023-07988-5 (PMC9850514; doi:10.1186/s12879-023-07988-5)
Supplement: Supplementary file 1 — Additional file 1. Full peer review history. [file 12879_2023_7988_MOESM1_ESM.docx]

**Full peer review history**

# Original submission

## Reviewer 1

This is a study on the causes of adult mortality (N=900+) in the Arba Minch Health and Demographic Surveillance Site, Ethiopia, from 2009 to 2017.Descriptions of the adult deaths (specific and broad causes of death were identified) were made by various socio-demographic characteristics.

This study is not without its large share of english gramar (and sentence construction) errors. For example in line 31: "73·4% of total deaths,". The manuscript needs a thorough english gramar check, ideally by an english native or a proofreading company.

Referencing also needs a strong overhaul as authors do not cite the correct studies. For exemple, authors cite "Karthika M, Philip S, Prathibha M, Varghese A, Rakesh P. Why are people dying due to tuberculosis? A study from Alappuzha District, Kerala, India. Indian Journal of Tuberculosis. 2018." To state that tuberculosis is the 9th cause of death worldwide.

In Methods, authors should better explain what were the procedures for the climatic zone and urbanization of the kebeles. Also please explain what a kebele is.

Please reference the WHO verbal autopsy questionnaire when first mentioned in methods

Figures do not have captions. I cannot tell which figure is which. Also I believe authors mention statistically significant diferences between male/female, however these should be highlighted in the table as well

## Reviewer 2

100% HIV and HBV co infection is a very unusual finding.
None of the patients were on ARV therapy
Why not. Had they ever been on ARV therapy in the past that would have driven resistance to HBV as ATRIPLA has been the standard of care for HIV treatment (which includes 3TC). It would be important to have details on HIV drug treatment experience, and also an explanation for why so many of these individuals were co infected, totally different from any other study done elsewhere.
Thus more information on the patients clinical characteristics, risk factors, previous antiretroviral therapy would be critical.
Information on HIV resistance in parallel with HCV resistance would complete the pieces of the puzzle.

## Response from author

Comment: This study is not without its large share of english gramar (and sentence construction) errors. For example in line 31: "73·4% of total deaths,". The manuscript needs a thorough english gramar check, ideally by an english native or a proofreading company.
Response: We thank the reviewer for the thoughtful and constructive comments given on our manuscript. The comments were so helpful, and we have revised the manuscript accordingly. We have now tried to correct the English grammar and sentence construction errors as much as possible manually and by online proof-reading platforms, according to your comments. The changes are highlighted in the revised manuscript through track changes.

Comments: Referencing also needs a strong overhaul as authors do not cite the correct studies. For exemple, authors cite "Karthika M, Philip S, Prathibha M, Varghese A, Rakesh P. Why are people dying due to tuberculosis? A study from Alappuzha District, Kerala, India. Indian Journal of Tuberculosis. 2018." To state that tuberculosis is the 9th cause of death worldwide.
Response: Thank you again for the valuable comment here. It is correct that we cited a reference of reference not the original reference wrongly. We have now corrected and cited the correct original reference for this specific example, and also, we have checked all the other references.

Comments: In Methods, authors should better explain what were the procedures for the climatic zone and urbanization of the kebeles. Also please explain what a kebele is.
Response: Thank you for these comments.
The procedures for climatic zone and urbanization of the kebeles, were not actually conducted by the authors. However, when the HDSS was established, sample of kebeles were taken from each climatic zone (i.e. Highland, Midland, Lowland), semi-urban and rural areas based on the status from the district (Arba Minch Zuria), to ensure representativeness. Arba Minch Zuria District had 31 kebeles where 29 are rural and 2 are semi urban. Besides it has three climatic zones classified as lowland, midland and highland.
Definition of kebele is now given in the methods section, as the smallest administrative units under the districts of Ethiopia.

Comment: Please reference the WHO verbal autopsy questionnaire when first mentioned in methods
Response: Thank you for this comment. We have referenced it now accordingly.

Comments: Figures do not have captions. I cannot tell which figure is which. Also I believe authors mention statistically significant diferences between male/female, however these should be highlighted in the table as well
Response: Thank you for these comments.
It is correct that the figure captions/legends are not given immediately after the figures. We did this as per the protocol of the journal. The captions/legends of the figures are listed at the end of the document after the references under the heading “Figure legends”.
Actually, there is no statistically significant differences between male/female regarding the broad causes of death (χ2 =3.4, P-value= 0.2) and trend of death by tuberculosis (Male: χ2 test for trend =1.24, P-value=0.27, Female: χ2 test for trend=2.61, P-value=0.11), as it is given under the figure legends.

# Revision 1

## Reviewer 3

This is an informative study of cause of adult mortality in Arba Minch in Ethiopia. It is unique in that it presents the Verbal autopsy findings from a low to lower middle income country. This is useful for other researchers. The finding are also quite interesting and the main cause of mortality is different fro Higher income countries and countries with less rural populations.


Abstract - Full Stop at end of Background.

Main Article
Background

Decreasing adult mortality rates are evidenced in studies from Korea, - Clarify if South Korea or North Korea.

Methods

Lines 55 to 58 - Kebele is the smallest administrative unit under the districts/woredas in Ethiopia. Suggest districts or woredas. Additionally use districts which are termed woredas. This will be clearer to international readers.
Lines 39 to 41 - next of families - This should be next of kin.
Lines 8 to 11 - But for, we categorized the death years into eight equal categories (i.e. one year) as follow - This needs to be clarified after "But for"

Under Results
Lines 10 to 12 - There was a single death assumed to be attributed due to misadventure to a patient during surgical
and medical care - consider another word other than misadventure (also in figure 1).

Broad comment on Figures - It would be clearer to the reader if each Figure was clearly labelled figure 1, 2, 3 etc. This would be similar to how tables are labelled. Noted at the end of References, however.

Discussion

General Comment - It is unnecessary to repeat percentages given in results. Just give a trend or interpretation.
Line 31 to 35 - This
indicates that tuberculosis is still the single killer of Ethiopian adults. - This should be reworded from single killer to cause of mortality.

Referencing

Generally, if Vancouver format is used Italics and shortening of the name or abbreviated name of the journal is the ideal method. In addition, if there are more than 6 authors the first 3 should be used followed by et al. However after reviewing publications of the journal, I do not see the current referencing as a problem.

## Reviewer 4

The study made 2009-2017. Add the 2017-2020.
Has ethical approval been obtained for the study?

## Reviewer 5

1. The introduction is long. Why is there mention of tuberculosis in the introduction when the study is to find the common cause of mortality among the adults was research question
2. There are lot of spelling mistakes for example communicable has been spelt differently each time it is used in the explanation or in the figures.
3. The discussion is not convincing. Each of the results need to be analysed.

## Reviewer 6

The manuscript has grammatical error so please edit the errors. In addition to this I shared the other reviewer comments.

Response from author

Point by point response to Reviewer 3
This is an informative study of cause of adult mortality in Arba Minch in Ethiopia. It is unique in that it presents the Verbal autopsy findings from a low to lower middle-income country. This is useful for other researchers. The finding are also quite interesting and the main cause of mortality is different from Higher income countries and countries with less rural populations.
Response: We thank the reviewer for the thoughtful and constructive comments given on our manuscript. The comments were so helpful, and we have revised the manuscript accordingly.
Abstract - Full Stop at end of Background.
Response: Thank you again for the valuable comment here. We have corrected it accordingly.
Background
Decreasing adult mortality rates are evidenced in studies from Korea, - Clarify if South Korea or North Korea.
Response: Thank you for this comment. We have now clarified as South Korea.
Methods
Lines 55 to 58 - Kebele is the smallest administrative unit under the districts/woredas in Ethiopia. Suggest districts or woredas. Additionally, use districts which are termed woredas. This will be clearer to international readers.
Response: Thank you for the important comments provided here in the Methods. Accordingly, we have corrected as follow: We have used the term districts consistently as per your suggestions.
Lines 39 to 41 - next of families - This should be next of kin.
Response : Corrected accordingly
Lines 8 to 11 - But for, we categorized the death years into eight equal categories (i.e. one year) as follow - This needs to be clarified after "But for"
Response: We have now clarified the statement as follow; “But for the purpose of analysis and comparison, we categorized the death years into eight equal categories (i.e. one year) as follow;”
Under Results
Lines 10 to 12 - There was a single death assumed to be attributed due to misadventure to a patient during surgical and medical care - consider another word other than misadventure (also in figure 1).
Response: Thank you for these comments. We used the word “misadventure” according to the WHO Verbal Autopsy standards and ICD-10 codes.
Broad comment on Figures - It would be clearer to the reader if each Figure was clearly labelled figure 1, 2, 3 etc. This would be similar to how tables are labelled. Noted at the end of References, however.
Response: Thank you very much for this comment. We had also received similar comment from another respected reviewer, and we have no problem of doing that. However, we wanted to stick to the guidelines of the journal. The captions/legends of the figures are listed at the end of the document after the references under the heading “Figure legends” as per the guideline/protocol of the journal.
Discussion
General Comment - It is unnecessary to repeat percentages given in results. Just give a trend or interpretation.
Response: Thank you also for the vital comments given in the discussion.
Accordingly, we have now tried to incorporate your comments and re-modify it. However, we put both the results and their interpretations only for the significant/important in the revised manuscript.
Line 31 to 35 - This indicates that tuberculosis is still the single killer of Ethiopian adults. - This should be reworded from single killer to cause of mortality.
Response : It is now reworded as single most cause of mortality, according to your comments.
Referencing
Generally, if Vancouver format is used Italics and shortening of the name or abbreviated name of the journal is the ideal method. In addition, if there are more than 6 authors the first 3 should be used followed by et al. However, after reviewing publications of the journal, I do not see the current referencing as a problem.
Response: We are also very thankful for the suggestions you have on the referencing format.
It is correct that the ideal methods are those you mentioned. We have used an EndNote referencing manager using Vancouver referencing style automatically.
Point by point response to Reviewer 4
The study made 2009-2017. Add the 2017-2020.
Response: We thank the reviewer for the thoughtful and constructive comments given on our manuscript.
It would be good if we can add the 2017-2020 data to the manuscript. However, permission to use the data was maintained earlier for the specified time period (i.e. 2009-2017), and will be difficult to add the remaining data due to the data sharing policy of the surveillance center. This is acknowledged as a limitation.
Has ethical approval been obtained for the study?
Response: Thanks again for this question.
Ethical approval has been obtained from Arba Minch University ethical review committee, Ethiopian public health association (EPHA) ethical review board, and the American Center for Disease Control (CDC) Atlanta during establishment of the Health and Demographic Surveillance site, as it is stated under the “Ethics approval and consent to participate”.
Therefore, we extracted the necessary data from the database after getting the necessary procedures and permissions from the concerned bodies of the HDSS.
Point by point response to Reviewer 5
The introduction is long. Why is there mention of tuberculosis in the introduction when the study is to find the common cause of mortality among the adults was research question
Response: We thank the reviewer for the thoughtful and constructive comments given on our manuscript. The comments were so helpful, and we have revised the manuscript accordingly.
We accepted the thoughtful comment provided here. Hence, we tried to shorten the introduction by omitting the paragraphs related to tuberculosis, as it is subset of the research question.
There are lot of spelling mistakes for example communicable has been spelt differently each time it is used in the explanation or in the figures.
Response: Thanks again for the important comment.
We have checked and corrected some spelling errors both in the explanations and figures.
The discussion is not convincing. Each of the results need to be analysed.
Response: Thank you again for your constructive comments here.
We have now tried to incorporate your comments and re-modify it. We have tried to analyze and discuss each of the important results.
Point by point response to Reviewer 6
The manuscript has grammatical error so please edit the errors. In addition to this I shared the other reviewer comments.
Response: We thank the reviewer for the thoughtful and constructive comments given on our manuscript. The comments were so helpful; thus, we have revised the manuscript accordingly.
We have tried to correct the English grammar and sentence construction errors as much as possible manually and by online proof-reading platforms, according to your comments.

# Revision 2

## Reviewer 7

This is an important piece of the study describing adult mortality causes using verbal autopsy data. However, the manuscript needs further modification to make it more informative especially in the background and other parts. I try to mention below.
1. Title: the title is long and can be rephrased as "Causes and trends of adult mortality in Southern Ethiopia: An eight-year follow-up database study"
Abstract
Abstract
1. line 48 " verbal autopsy method site" to verbal autopsy method
2. Line 8, what is the external causes of death (13.5%)? confusing in the abstract part
3. Line 11-12 "……leading causes of adult mortality yielding higher adult mortality" rephrases as "leading causes of adult mortality"
Background
1. The background is not focused on your study question and difficult to grasp the concept. It can be organized as
­ What is the adult overall mortality rate using recent data
­ What trend showed in causes of death, Communicable Vs Noncommunicable
­ Is the cause of adult Mortality has a similar trend in developed Vs low-income countries,
­ What has been seen in Ethiopia with a change in cause of death in Ethiopia
2. You used old data while recent available such as globally, approximately 56.9 million people died in 2016.
3. Repeated similar concepts. E.g paragraph 1 and 2 is similar except the year. Such explanation should be rephrased and combined

Methods
1. Is semi-urban similar to urban kebele? pleases make consistent
2. Why 8 rural and only one semi-Urban/ urban study site. Such selection skewed the pattern of the cause of death. E.g communicable vs Non- communicable vs Urban Vs Rural
3. Data Collection Procedures: Add the Verbal autopsy (VA) coding and the collection toll you used as an annex
Result
1. Causes of Adult Deaths: This subtitle should be revised. There are causes of death described rather just description of the death recording.
2. Broad causes: In this subtitle first describe the cause of death as Communicable, non-communicable and external course then more specific cause as infectious, parasitic………….. just rearrange paragraph one and two in this subtitle
3. Line 56; however, a nearly identical trend was observed for both males and females though it is significant. This sentence is confusing. Pleases rephrase it
4. It is good to add, the figure showed the trend of communicable Vs. Non-communicable Vs external Vs other. This is more important in terms of intervention
5. Figure 5, not use the year1,2,3 in capital letter


Discussion
­ Conclusion: since your study did not refer to the source population in each study year and about 90% of your study is in the rural area, concluding about the predominant of communicable disease, actually expected, is skewed due to your sampling. Please be cautious in this regard.

## Reviewer 8

Major Observations (must be corrected)

Page 04 Line 45 Abstract/Methods: Say full dates 2009-Sep-12 to 2017-Sep-11.
Page 05 Line 04 Abstract/Results: Say 'or', not 'and'.
Page 05 Line 49 Background: Check sources and units, the 10.9 figure does not appear in the #5 reference given, does not agree with previous paragraphs.
Page 06 Line 32 Methods/Site: Provide references describing Arba Minch HDSS (#20 is not a valid nor useful reference).
Page 06 Line 36-40 Methods/Site: Correct, description does not match with sampling, strata or clusters. It is good enough to say that 9 rural + 1 urban kebeles (adm units below districts) were intentionally selected as catchment area.
Page 07 Line 16 Methods/Data: Clarify. What do you mean by "usually".
Page 09 Line 16 Results: Present data on number and reasons for deaths excluded from analysis (are those in page 10?), also for people lost to follow-up as well as immigrants, if any. Present data on denominator population over time.
Page 09 Table 1 Results: Provide another column with those descriptions for the overall populatiuon under surveillance.
Page 14 Figures Results: Provide numbers, the trend in proportional tb deaths is not consistent with the trend in tb mortality. Peaks at years 1 and 8 maybe methodological.
Page 16 Limitations: Discuss representativeness of study area and tour (probably grounded) basis por suggesting extrapolation.
Page 16 Limitations: Discuss biases originated by (local or greater) migrations and refusals to participate.
Page 16 Limitations: Discuss specific limitations for verbal dx of tb (expected sensitivity and specificity for instance).
Page 16 Line 23 Conclusions: revise phrases according to your discussion of limitations.
